# Supplementary figures and images for: Factors associated with hepatocellular carcinoma occurrence after HCV eradication in patients without cirrhosis or with compensated cirrhosis
Source: PLoS One. 2020 Dec 7;15(12):e0243473. doi: 10.1371/journal.pone.0243473 (PMC7721183; doi:10.1371/journal.pone.0243473)

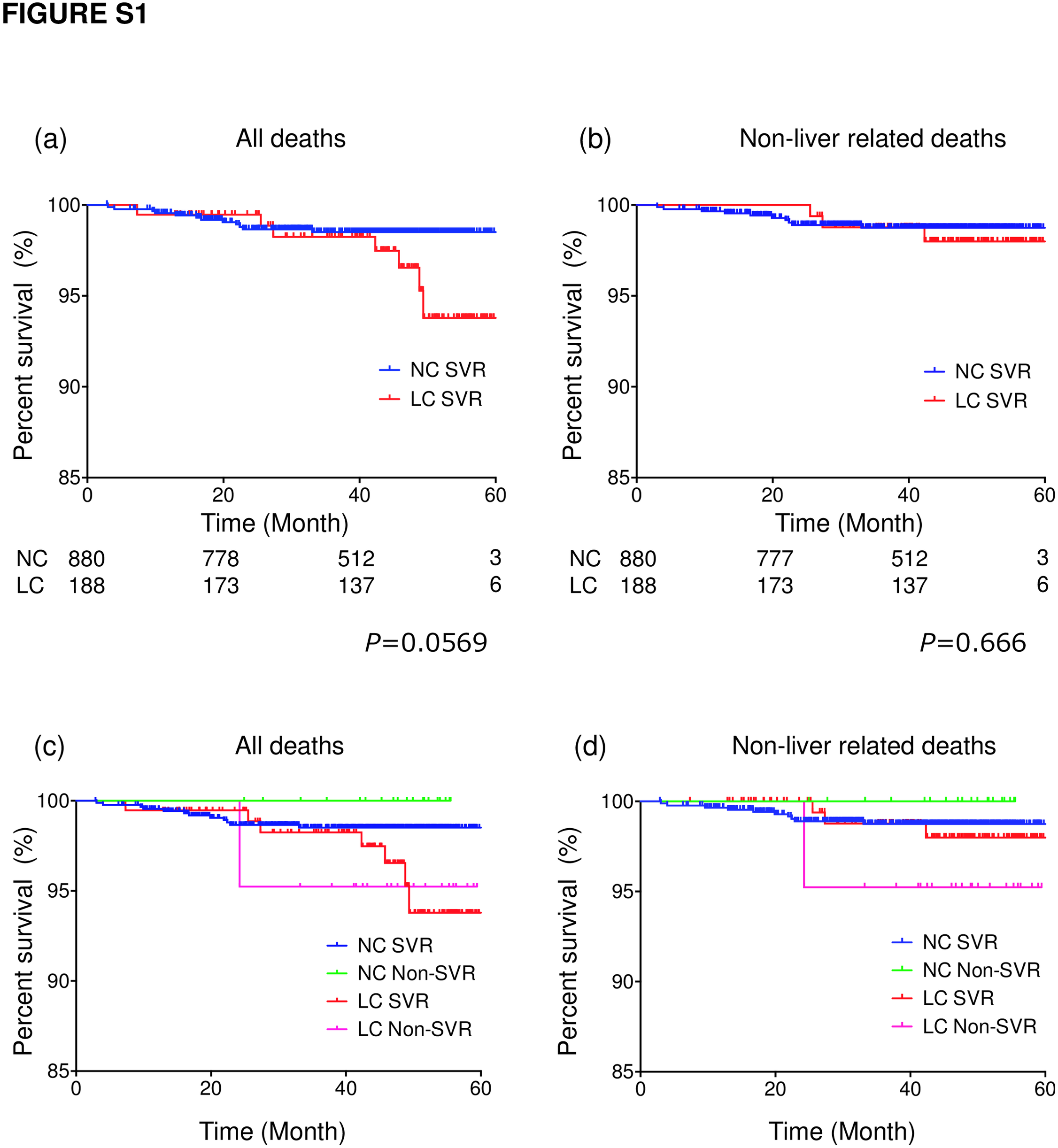

Supplement: S1 Fig — (a) All survival rates of patients with compensated liver cirrhosis (red line) and patients without cirrhosis (blue line). (b) Non-liver-related survival rates of patients with compensated liver cirrhosis (red line) and patients without cirrhosis (blue line). (c) All survival rates of SVR patients with compensated liver cirrhosis (red line), non-SVR patients with compensated liver cirrhosis (pink line), SVR patients without cirrhosis (blue line), and non-SVR patients without cirrhosis (green line). (d) The non-liver-related survival rates of SVR patients with compensated liver cirrhosis (red line), non-SVR patients with compensated liver cirrhosis (pink line), SVR patients without cirrhosis (blue line), and non-SVR patients without cirrhosis (green line). The results were analyzed with the log-rank test. (TIF) [file pone.0243473.s001.tif]

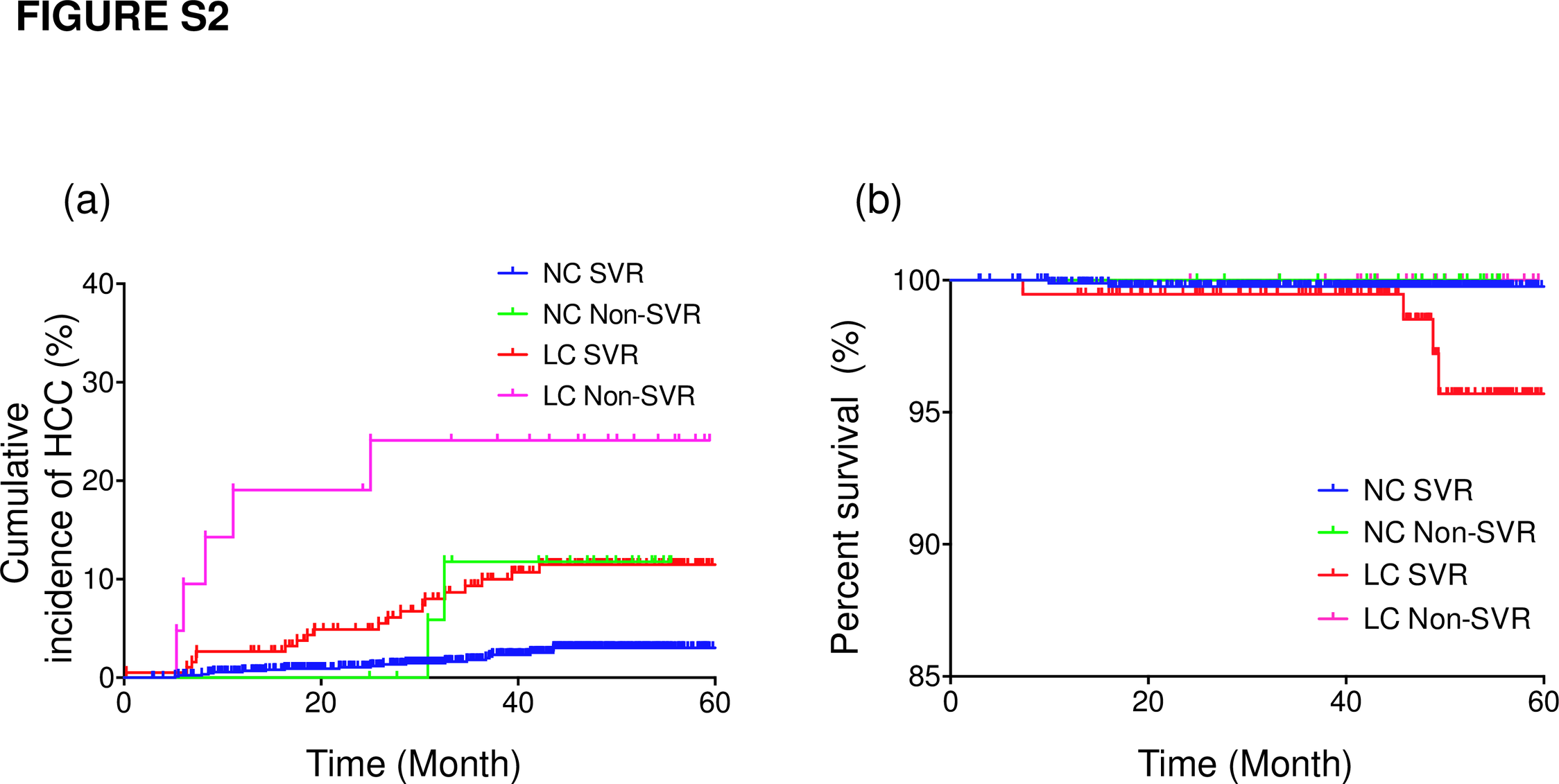

Supplement: S2 Fig — (a) Cumulative incidence of hepatocellular carcinoma in SVR patients with compensated liver cirrhosis (red line), non-SVR patients with compensated liver cirrhosis (pink line), SVR patients without cirrhosis (blue line), and non-SVR patients without cirrhosis (green line). (b) The liver-related survival rate of SVR patients with compensated liver cirrhosis (red line), non-SVR patients with compensated liver cirrhosis (pink line), SVR patients without cirrhosis (blue line), and non-SVR patients without cirrhosis (green line). The results were analyzed using the log-rank test. (TIF) [file pone.0243473.s002.tif]

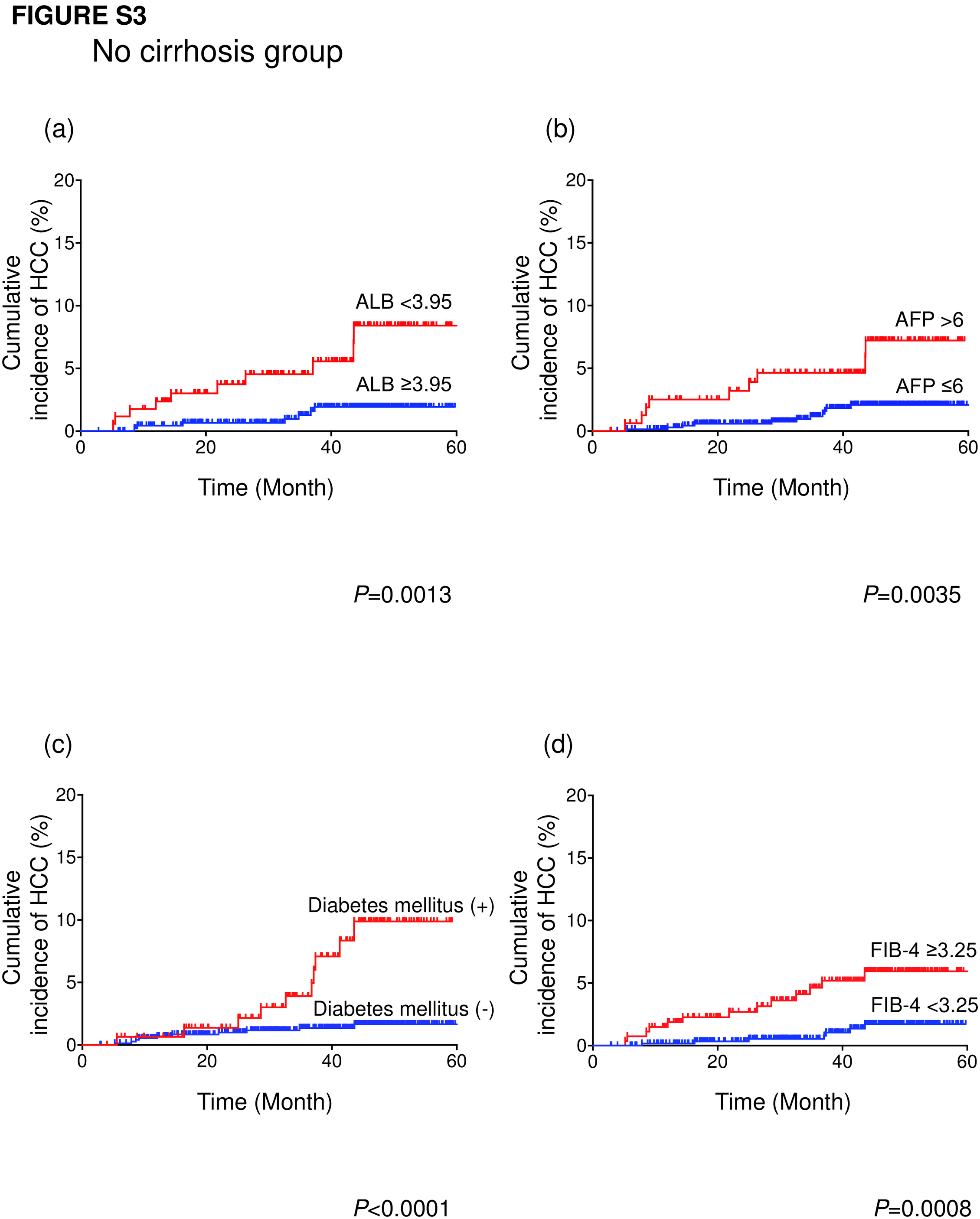

Supplement: S3 Fig — (a) Serum ALB level of <3.95 g/dl (red line); serum ALB level of ≥3.95 g/dl (blue line). (b) Serum AFP level of >6 ng/ml (red line); serum AFP level of ≤6 ng/ml (blue line). (c) Presence of DM before DAA treatment (red line); absence of DM (blue line). (d) FIB-4 score of ≥3.25 (red line); FIB-4 score of <3.25 (blue line). The results were analyzed using the log-rank test. (TIF) [file pone.0243473.s003.tif]

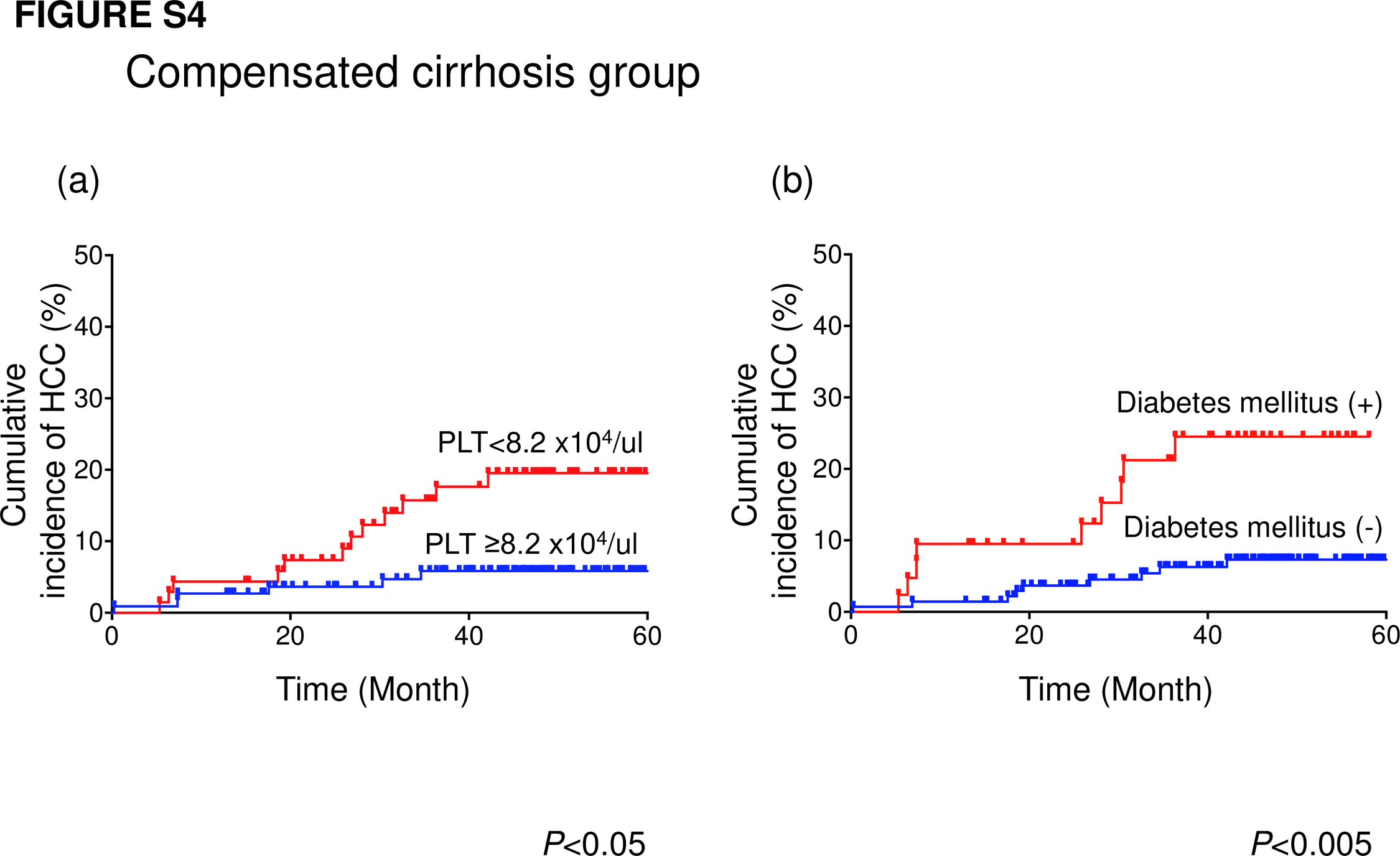

Supplement: S4 Fig — (a) The PLT, <8.2 x10^4^/μl (red line), ≥8.2 x10^4^ (blue line). (b) The presence of DM before DAA treatment (red line), absence of DM (blue line). Results were analyzed by log-rank test. (TIF) [file pone.0243473.s004.tif]
