# Supplementary material for: Factors associated with hepatocellular carcinoma occurrence after HCV eradication in patients without cirrhosis or with compensated cirrhosis
Source: PLoS One. 2020 Dec 7;15(12):e0243473. doi: 10.1371/journal.pone.0243473 (PMC7721183; doi:10.1371/journal.pone.0243473)
Supplement: S1 Table — (DOCX) [file pone.0243473.s005.docx]

**S1 Table.** Characteristics of 1108 patients with hepatitis C virus (included 40 patients with non-SVR)

|  | All  (n=1108) | No cirrhosis  (n=899) | Compensated  cirrhosis  (n=209) | *P*-value |
| --- | --- | --- | --- | --- |
| Age, years, median (IQR) | 67 (57-75) | 66 (56-74) | 70 (61-77) | <0.0001* |
| Sex, male/female  (male %) | 529/579  (48%) | 432/467  (48%) | 97/112  (46%) | 0.6686 |
| Genotype, 1/2/other  (G-1%) | 746/354/8  (67%) | 598/294/7  (67%) | 148/60/1  (71%) | 0.2189 |
| HCV RNA, LogIU/ml, median (IQR) | 6.2 (5.6-6.6) | 6.2 (5.7-6.6) | 6.1 (5.5-6.5) | 0.0627 |
| History of interferon-based therapy, yes (%) | 326  (29%) | 257  (29%) | 69  (33%) | 0.2058 |
| Diabetes mellitus, n  (%) | 205  (19%) | 156  (17%) | 49  (23%) | 0.0795 |
| HBcAb positive, n  (%) | 64  (6%) | 53  (6%) | 11  (5%) | 0.7241 |
| SVR, n (%) | 1068 (96%) | 880 (98%) | 188 (90%) | <0.0001* |
| Observation period after DAA treatment, months, median (IQR) | 43 (30-49) | 42 (31-48) | 45 (32-51) | 0.0011* |
| ALB, g/dl, median (IQR) | 4.1 (3.8-4.4) | 4.2 (3.9-4.4) | 3.8 (3.6-4.1) | <0.0001* |
| TB, mg/dl, median (IQR) | 0.8 (0.6-1.0) | 0.8 (0.6-0.9) | 0.9 (0.7-1.1) | <0.0001* |
| AST, U/l, median (IQR) | 40 (27-60) | 37 (26-53) | 53 (38-76) | <0.0001* |
| ALT, U/l, median (IQR) | 38 (26-62) | 36 (25-60) | 47 (32-76) | <0.0001* |
| GGT, U/l, median (IQR) | 32 (20-58) | 31 (19-57) | 39 (25-62) | 0.0016* |
| eGFR, ml/min/1.73 m^2^, median (IQR) | 72 (62-83) | 72 (62-83) | 71 (58-82) | 0.2508 |
| PLT, ×10^4^/µl, median (IQR) | 15.5 (11.8-19.7) | 16.9 (13.6-20.8) | 9.0 (7.1-11.1) | <0.0001* |
| FIB-4 score, median (IQR) | 2.8 (1.8-4.5) | 2.5 (1.6-3.6) | 6.1 (4.4-8.7) | <0.0001* |
| ALBI score, median (IQR) | -2.8 (-3.0- -2.5) | -2.8 (-3.0- -2.6) | -2.5 (-2.8- -2.2) | <0.0001* |
| AFP, ng/ml, median (IQR) | 4.1 (2.6-7.3) | 3.6 (2.5-5.9) | 8.7 (4.3-20.3) | <0.0001* |

**P* < 0.05 was considered significant (no cirrhosis vs compensated cirrhosis).

Abbreviations: DAA, direct-acting antiviral; HCC, hepatocellular carcinoma; ALB, albumin; TB, total bilirubin; AST, aspartate aminotransferase; ALT, alanine aminotransferase; GGT, γ-glutamyltransferase; PLT, platelet count; FIB-4, fibrosis-4; ALBI, albumin–bilirubin; AFP, α-fetoprotein; IQR, interquartile range; SVR, sustained viral response.
