# Supplementary material for: Factors associated with hepatocellular carcinoma occurrence after HCV eradication in patients without cirrhosis or with compensated cirrhosis
Source: PLoS One. 2020 Dec 7;15(12):e0243473. doi: 10.1371/journal.pone.0243473 (PMC7721183; doi:10.1371/journal.pone.0243473)
Supplement: S2 Table — (DOCX) [file pone.0243473.s006.docx]

**S2 Table.** Pretreatment factors associated with the development of hepatocellular carcinoma after DAA treatment in HCV-positive patients without cirrhosis (included 19 patients with non-SVR)

|  | No HCC  (n=877) | HCC  (n=22) | *P*-value |
| --- | --- | --- | --- |
| Age, years, median (IQR) | 66 (56-74) | 74 (68-76) | 0.0020* |
| Sex, male/female (male %) | 417/460 (48%) | 15/7 (68%) | 0.0557 |
| Genotype, 1/2/other (G-1%) | 580/290/7  (66%) | 18/4/0  (82%) | 0.1197 |
| HCV RNA, LogIU/ml, median (IQR) | 6.2 (5.7-6.6) | 6.0 (5.5-6.2) | 0.0175* |
| History of interferon-based therapy, yes (%) | 248  (28%) | 9  (41%) | 0.1953 |
| Diabetes mellitus, n (%) | 146 (17%) | 10 (45%) | 0.0004* |
| HBcAb positive, n (%) | 49 (6%) | 4 (18%) | 0.0132* |
| SVR, n (%) | 860 (98%) | 20 (91%) | 0.0212* |
| Observation period after DAA treatment, months, median (IQR) | 42 (31-48) | 46 (40-49) | 0.1036 |
| ALB, g/dl, median (IQR) | 4.2 (3.9-4.4) | 3.8 (3.7-4.2) | 0.0004* |
| TB, mg/dl, median (IQR) | 0.8 (0.6-0.9) | 0.8 (0.6-1.0) | 0.5686 |
| AST, U/l, median (IQR) | 36 (26-53) | 49 (44-57) | 0.0278* |
| ALT, U/l, median (IQR) | 36 (25-59) | 46 (35-60) | 0.2019 |
| GGT, U/l, median (IQR) | 31 (19-57) | 35 (20-52) | 0.9596 |
| eGFR, ml/min/1.73 m^2^, median (IQR) | 72 (62-83) | 77 (72-83) | 0.3127 |
| PLT, ×10^4^/µl, median (IQR) | 17.0 (13.8-21.0) | 12.8 (11.0-16.6) | 0.0002* |
| FIB-4 score, median (IQR) | 2.4 (1.6-3.6) | 4.2 (3.1-5.1) | <0.0001* |
| ALBI score, median (IQR) | -2.8 (-3.0- -2.6) | -2.6 (-2.9- -2.3) | 0.0011* |
| AFP, ng/ml, median (IQR) | 3.5 (2.5-5.8) | 6.2 (3.6-7.9) | 0.0045* |

**P* < 0.05 was considered significant (no HCC vs HCC).

Abbreviations: DAA, direct-acting antiviral; HCC, hepatocellular carcinoma; ALB, albumin; TB, total bilirubin; AST, aspartate aminotransferase; ALT, alanine aminotransferase; GGT, γ-glutamyltransferase; PLT, platelet count; FIB-4, fibrosis-4; ALBI, albumin–bilirubin; AFP, α-fetoprotein; IQR, interquartile range; SVR, sustained viral response.
