# Supplementary material for: Factors associated with hepatocellular carcinoma occurrence after HCV eradication in patients without cirrhosis or with compensated cirrhosis
Source: PLoS One. 2020 Dec 7;15(12):e0243473. doi: 10.1371/journal.pone.0243473 (PMC7721183; doi:10.1371/journal.pone.0243473)
Supplement: S3 Table — (DOCX) [file pone.0243473.s007.docx]

**S3 Table.** Pretreatment factors associated with the development of HCC after DAA treatment in HCV-positive patients with compensated liver cirrhosis (includes 21 patients with non-SVR)

|  | No HCC  (n=185) | HCC  (n=24) | *P*-value |
| --- | --- | --- | --- |
| Age, years, median (IQR) | 71 (60-77) | 70 (66-74) | 0.9240 |
| Sex, male/female (male %) | 81/104  (44%) | 16/8  (67%) | 0.0344* |
| Genotype, 1/2/other (G-1%) | 128/56/1  (69%) | 20/4/0  (84%) | 0.1474 |
| HCV RNA, LogIU/ml, median (IQR) | 6.1 (5.5-6.5) | 6.1 (5.6-6.3) | 0.5438 |
| History of interferon-based therapy, yes (%) | 58  (31%) | 11  (46%) | 0.1558 |
| Diabetes mellitus, n (%) | 39 (21%) | 10 (42%) | 0.0251* |
| HBcAb positive, n (%) | 8 (4%) | 3 (13%) | 0.0915 |
| SVR, n (%) | 169 (91%) | 19 (79%) | 0.0618 |
| Observation period after DAA treatment, months, median (IQR) | 46 (38-53) | 19 (7-30) | <0.0001* |
| ALB, g/dl, median (IQR) | 3.9 (3.6-4.1) | 3.6 (3.3-3.9) | 0.0043* |
| TB, mg/dl, median (IQR) | 0.8 (0.6-1.1) | 1.1 (0.8-1.4) | 0.0106* |
| AST, U/l, median (IQR) | 53 (38-77) | 52 (39-62) | 0.6282 |
| ALT, U/l, median (IQR) | 46 (32-78) | 49 (29-71) | 0.7435 |
| GGT, U/l, median (IQR) | 38 (25-60) | 39 (26-71) | 0.8146 |
| eGFR, ml/min/1.73 m^2^ , median (IQR) | 71 (60-83) | 67 (56-80) | 0.7656 |
| PLT, ×10^4^/µl, median (IQR) | 9.2 (7.2-11.4) | 8.1 (6.4-9.2) | 0.0387* |
| FIB-4 score, median (IQR) | 6.0 (4.4-8.6) | 6.9 (4.9-9.3) | 0.2431 |
| ALBI score, median (IQR) | -2.5 (-2.8- -2.3) | -2.2 (-2.5- -1.9) | 0.0007* |
| AFP, ng/ml, median (IQR) | 8.0 (4.2-20.3) | 16.6 (6.6-29.3) | 0.0686 |

**P* < 0.05 was considered significant (no HCC vs HCC).

Abbreviations: DAA, direct-acting antiviral; HCC, hepatocellular carcinoma; ALB, albumin; TB, total bilirubin; AST, aspartate aminotransferase; ALT, alanine aminotransferase; GGT, γ-glutamyltransferase; PLT, platelet count; FIB-4, fibrosis-4; ALBI, albumin–bilirubin; AFP, α-fetoprotein; IQR, interquartile range.
