# Supplementary material for: Factors associated with hepatocellular carcinoma occurrence after HCV eradication in patients without cirrhosis or with compensated cirrhosis
Source: PLoS One. 2020 Dec 7;15(12):e0243473. doi: 10.1371/journal.pone.0243473 (PMC7721183; doi:10.1371/journal.pone.0243473)
Supplement: S4 Table — (DOCX) [file pone.0243473.s008.docx]

**S4 Table.** Factors associated with the development of HCC after DAA treatment in HCV-positive patients without cirrhosis by Cox univariate analysis

| HCC vs  No HCC |  | Univariate | |
| --- | --- | --- | --- |
|  |  |  |  |
|  |  | HR (95% CI) | *P* |
| Age | ≥65 | 4.11 (1.37-17.61) | 0.0093* |
|  | <65 | 1 (Ref) |  |
| Male | Yes | 2.80 (1.12-7.93) | 0.0268* |
|  | No | 1 (Ref) |  |
| ALB (g/dl) | <3.95 | 4.28 (1.64-11.8) | 0.0032* |
|  | ≥3.95 | 1 (Ref) |  |
| AFP (ng/ml) | >6 | 3.72 (1.42-9.91) | 0.0082* |
|  | ≤6 | 1 (Ref) |  |
| DM | Yes | 5.02 (2.06-12.25) | 0.0006* |
|  | No | 1 (Ref) |  |
| FIB-4 score | ≥3.25 | 4.10 (1.68-10.93) | 0.0019* |
|  | <3.25 | 1 (Ref) |  |

Ref, reference group; HR, hazard ratio; CI, confidence interval.

**P* < 0.05 was considered significant.

Abbreviations: HCC, hepatocellular carcinoma; AFP, α-fetoprotein; ALB, albumin; DM, diabetes mellitus; FIB-4, fibrosis-4.
