# Supplementary material for: Factors associated with hepatocellular carcinoma occurrence after HCV eradication in patients without cirrhosis or with compensated cirrhosis
Source: PLoS One. 2020 Dec 7;15(12):e0243473. doi: 10.1371/journal.pone.0243473 (PMC7721183; doi:10.1371/journal.pone.0243473)
Supplement: S5 Table — (DOCX) [file pone.0243473.s009.docx]

**S5 Table**. Post-treatment factors associated with the development of hepatocellular carcinoma after DAA treatment in HCV-positive patients without cirrhosis

|  | No HCC  (n=860) | HCC  (n=20) | *P*-value |
| --- | --- | --- | --- |
| ALB, g/dl, median (IQR) | 4.3 (4.1-4.5) | 4.1 (4.1-4.2) | 0.0346* |
| TB, mg/dl, median (IQR) | 0.7 (0.6-0.9) | 0.8 (0.6-1.0) | 0.6865 |
| AST, U/l, median (IQR) | 22 (18-26) | 29 (25-33) | 0.0006* |
| ALT, U/l, median (IQR) | 15 (12-21) | 20 (15-29) | 0.0727 |
| GGT, U/l, median (IQR) | 19 (14-28) | 25 (16-43) | 0.1920 |
| eGFR, ml/min/1.73 m^2^, median (IQR) | 68 (59-79) | 74 (63-82) | 0.2264 |
| PLT, ×10^4^/µl, median (IQR) | 18.3 (14.8-22.7) | 12.7 (10.0-18.5) | 0.0018* |
| FIB-4 score, median (IQR) | 2.0 (1.3-2.9) | 3.3 (2.8-4.6) | <0.0001* |
| ALBI score, median (IQR) | -2.9 (-3.1- -2.7) | -2.7 (-2.8- -2.6) | 0.0177* |
| AFP, ng/ml, median (IQR) | 2.8 (2.0-3.7) | 4.8 (3.5-6.8) | 0.0005* |

**P* < 0.05 was considered significant (no HCC vs HCC).

Abbreviations: DAA, direct-acting antiviral; HCC, hepatocellular carcinoma; ALB, albumin; TB, total bilirubin; AST, aspartate aminotransferase; ALT, alanine aminotransferase; GGT, γ-glutamyltransferase; PLT, platelet count; FIB-4, fibrosis-4; ALBI, albumin–bilirubin; AFP, α-fetoprotein; IQR, interquartile range.
