# Supplementary material for: Factors associated with hepatocellular carcinoma occurrence after HCV eradication in patients without cirrhosis or with compensated cirrhosis
Source: PLoS One. 2020 Dec 7;15(12):e0243473. doi: 10.1371/journal.pone.0243473 (PMC7721183; doi:10.1371/journal.pone.0243473)
Supplement: S6 Table — (DOCX) [file pone.0243473.s010.docx]

**S6 Table**. Post-treatment factors associated with the development of HCC after DAA treatment in HCV-positive patients without cirrhosis by Cox multivariate model

| HCC vs  No HCC |  | Model 1^a^ | |  | Model 2^b^ | |
| --- | --- | --- | --- | --- | --- | --- |
|  |  |  |  |  |  |  |
|  |  | HR (95% CI) | *P* |  | HR (95% CI) | *P* |
| ALB (g/dl) | <3.95 |  |  |  | 1.19 (0.26-4.22) | 0.7998 |
|  | ≥3.95 |  |  |  | 1 (Ref) |  |
| AFP (ng/ml) | >6 |  |  |  | 5.70 (1.47-19.34) | 0.0147* |
|  | ≤6 |  |  |  | 1 (Ref) |  |
| DM | Yes |  |  |  | 1.26(0.26-4.69) | 0.7493 |
|  | No |  |  |  | 1 (Ref) |  |
| FIB-4 score | ≥3.25 | 3.94 (1.48-10.75) | 0.0068* |  | 6.01 (1.60-26.94) | 0.0079* |
|  | <3.25 | 1 (Ref) |  |  | 1 (Ref) |  |

Ref, reference group; HR, hazard ratio; CI, confidence interval.

^a^After adjusting for age and sex. ^b^After adjusting for age, sex, AFP, DM and FIB-4 score.

**P* < 0.05 was considered significant.

Abbreviations: HCC, hepatocellular carcinoma; AFP, α-fetoprotein; ALB, albumin; DM, diabetes mellitus; FIB-4, fibrosis-4.
