# Supplementary material for: Factors associated with hepatocellular carcinoma occurrence after HCV eradication in patients without cirrhosis or with compensated cirrhosis
Source: PLoS One. 2020 Dec 7;15(12):e0243473. doi: 10.1371/journal.pone.0243473 (PMC7721183; doi:10.1371/journal.pone.0243473)
Supplement: S7 Table — (DOCX) [file pone.0243473.s011.docx]

**S7 Table.** Post-treatment factors associated with the development of HCC after DAA treatment in HCV-positive patients with compensated liver cirrhosis

|  | No HCC  (n=169) | HCC  (n=19) | *P* value |
| --- | --- | --- | --- |
| ALB, g/dl, median (IQR) | 4.2 (4.0-4.5) | 3.7 (3.4-4.1) | 0.0091* |
| TB, mg/dl, median (IQR) | 1.0 (0.8-1.3) | 1.0 (0.8-1.3) | 0.6636 |
| AST, U/l, median (IQR) | 27 (22-32) | 26 (20-30) | 0.6976 |
| ALT, U/l, median (IQR) | 18 (14-25) | 17 (15-32) | 0.5980 |
| GGT, U/l, median (IQR) | 23 (17-37) | 22 (17-33) | 0.9143 |
| eGFR, ml/min/1.73 m^2^, median (IQR) | 68 (59-76) | 64 (54-77) | 0.8654 |
| PLT, ×10^4^/µl, median (IQR) | 10.3 (7.6-13.3) | 8.2 (6.9-9.0) | 0.0015* |
| FIB-4 score, median (IQR) | 4.5 (3.1-5.8) | 5.2 (4.4-7.1) | 0.0589 |
| ALBI score, median (IQR) | -2.8 (-3.0- -2.6) | -2.4 (-2.7- -2.1) | 0.0205* |
| AFP, ng/ml, median (IQR) | 4.2 (2.8-5.9) | 6.6 (3.6-7.4) | 0.0408* |

**P* < 0.05 was considered significant (no HCC vs HCC).

Abbreviations: DAA, direct-acting antiviral; HCC, hepatocellular carcinoma; ALB, albumin; TB, total bilirubin; AST, aspartate aminotransferase; ALT, alanine aminotransferase; GGT, γ-glutamyltransferase; PLT, platelet count; FIB-4, fibrosis-4; ALBI, albumin–bilirubin; AFP, α-fetoprotein; IQR, interquartile range.
