# Supplementary material for: Factors associated with hepatocellular carcinoma occurrence after HCV eradication in patients without cirrhosis or with compensated cirrhosis
Source: PLoS One. 2020 Dec 7;15(12):e0243473. doi: 10.1371/journal.pone.0243473 (PMC7721183; doi:10.1371/journal.pone.0243473)
Supplement: S8 Table — (DOCX) [file pone.0243473.s012.docx]

**S8 Table.** Post-treatment factors associated with the development of HCC after DAA treatment in HCV-positive patients with compensated liver cirrhosis based on the Cox multivariate model

| HCC versus  No HCC |  | Model 1^a^ | |  | Model 2^b^ | |
| --- | --- | --- | --- | --- | --- | --- |
|  |  |  |  |  |  |  |
|  |  | HR (95% CI) | *P* |  | HR (95% CI) | *P* |
| ALBI score | >-2.3 | 4.99 (1.48-15.23) | 0.0114* |  | 5.42 (1.59-17.06) | 0.0089* |
|  | ≤-2.3 | 1 (Ref) |  |  | 1 (Ref) |  |
| DM | Yes |  |  |  | 1.99 (0.52-7.07) | 0.3018 |
|  | No |  |  |  | 1 (Ref) |  |
| PLT (×10^4^/μl) | <8.2 |  |  |  | 1.87 (0.56-6.17) | 0.2998 |
|  | ≥8.2 |  |  |  | 1 (Ref) |  |

Ref, reference group; HR, hazard ratio; CI, confidence interval.

^a^After adjusting for age and sex. ^b^After adjusting for age, sex, DM, and PLT.

**P* < 0.05 was considered significant.

Abbreviations: HCC, hepatocellular carcinoma; ALBI, albumin–bilirubin; DM, diabetes mellitus; PLT, platelet count.
